# Tooth polishing: Relevance in present day periodontal practice

**DOI:** 10.4103/0972-124X.51899

**Published:** 2009

**Authors:** Charu Madan, Rhythm Bains, Vivek K. Bains

**Affiliations:** *Senior Lecturer, Department of Periodontics, Manav Rachna Dental College, Faridabad, Haryana - 121 001, India*; 1 *Senior Lecturer, Department of Conservative Dentistry and Endodontics, India*; 2 *Assistant Professor, Department of Periodontics, Career Post Graduate Institute of Dental Sciences and Hospital, Lucknow, UP – 226 020, India*

**Keywords:** Air polisher device, polishing, porte polisher

## Abstract

Time has seen the emergence of more efficient and effective devices like jet abrasives. However, the role of rubber cups with prophy angles cannot be overlooked as they are still being widely used and provide an economical alternative. Owing to several shortcomings associated with the air polishing device using sodium bicarbonate (NaHCO_3_), trends are shifting towards the usage of low abrasive powders. Recent demonstration of Glycine Powder Air Polishing (GPAP) in removing subgingival biofilm results in less gingival erosion than hand instrumentation or NaHCO_3_ air-polishing. Despite the emergence of latest advances in polishing, data suggesting selective polishing of teeth is compelling.

In present day dental practice, polishing agents are used in stain removal as a “selective procedure”. Not every patient needs it, especially on regular basis, as continuous polishing over time causes morphological changes in teeth by abrading tooth structure, in addition to fluoride in outer enamel layer. During the initial or supportive periodontal therapy, polishing devices such as rotating rubber cups with polishing paste or air powder polishing devices are frequently used for professional supragingival plaque removal.[1] Port polisher consisting of orangewood points is helpful in situations when aerosol should not be produced, in abraded cervical areas, or when electricity is not available. However slowness of the procedure and amount of hand strength for instrumentation are its drawback. Although highly abrasive in nature, polishing or finishing strips present an option for interproximal areas or line angles but should be cautiously used to avoid cutting of soft tissues.[2] For over half a century, the most common method of tooth polishing is using rubber cup and pumice, and the widely used polishing paste consists of flour of pumice, glycerine, colour additive and in addition, sodium fluoride (NaF) or stannous fluoride (SnF_2_) might be added to this mix for desensitising effect. Other agents for polishing such as zinc oxide or kaolinite pastes, emery and tinoxy ore have also been suggested but fine pumice powder and glycerine paste or prophy paste enjoys maximum popularity, and recent use of perlite as a prophylaxis paste, make it a promising agent that might possess the abrasive properties of an ideal prophylaxis paste,[3] but aggressive use of rubber cup may remove a layer of cementum, which is thin in the cervical area. It is recommended to clean and heat sterilise all prophy angle and slow speed hand piece components used intra-orally between patients and in addition, disposable prophy angle serve as an alternative solution that eliminates the possibility of cross contamination inherent with sterilisation, from organic debris that survives it, though at some additional cost. While polishing with rubber cup, hand-piece used should be having sufficient torque to ensure steady speed (2500 rpm to 3000 rpm) that can easily be maintained. Empty cup will generate heat instead of polishing and one full rubber cup is sufficient for one or two teeth.[2] Least abrasive polishing paste should be used to remove stain with intermittent pressure that allows dissipation of heat, moreover inclusion of moisture throughout polishing reduces temperature rise during the process. Rotation speed, abrasiveness of paste, pressure applied with hand piece and duration influence the efficacy of polishing with rubber cup and prophylaxis paste.[1]

Nowadays, air powder polishing devices have overcome conventional rubber cup polishing paste systems for supragingival plaque removal as it reaches surfaces that are inaccessible to a rotary device.[1] The air powered abrasive machines used for prophylaxis in the past (Air Dent Machine) sprayed 30 μm alumina or dolomite particles at a pressure of 80 psi and at a velocity of 1900 ft./sec. Redesigned Air Polishing Device (APD), the Prophy-jet, works by directing pressurized slurry of sodium bicarbonate in warm water on to the tooth surface, at a suggested distance of 4-5 mm and angulation of around 60°.[4] Stream of abrasive particles being conveyed to the tooth surface by a non-abrasive vehicle and in case of the Prophy-jet the abrasive is NaHCO_3_ with Tri-Calcium Phosphate (added upto 0.8% of weight to improve flow characteristics) and the non-abrasive vehicle is a combination of water and air. Powder-water setting, the distance of the jet from the treated surface and the shape and size of the particles used control the effectiveness of the device.[1] It is a time saving, safe and effective for stain removal (also for smoking and chlorhexidine stain), thus minimize the operator and patient fatigue. However, cautious use of air polishers in patients with restricted sodium diets, with respiratory renal or metabolic disease, infectious diseases, children, on diuretics or long term steroid therapy, and with titanium implants has been advocated. Dentinal sensitivity is diminished following the use of Prophy-jet that may be explained by the fact that bicarbonate crystals may block the tubular openings and it has a unique ability to remove plaque from areas that are otherwise difficult to reach like furcations, flutings and close root proximities. APD is effective mean for removal of plaque from orthodontically banded and bracketed teeth as it does not disturb the wires or rubber bands and also is not detrimental to zinc phosphate or resin cements, used to attach brackets and bands, when the spray is directed at 90°.[5] Substantial reduction of gingival bleeding and marginal redness in orthodontic patients has been observed with air polishing method, but this system has the potential for removing considerable amount of resinous restorative material when used at recommended 60° although porcelain amalgam and gold alloy restorations are not significantly affected. It is useful in root detoxification during periodontal surgery however, APD with NaHCO_3_ may not be safely utilised on exposed root surfaces. A 30- second exposure of a fixed point on a root surface to a Prophy-jet action produced crater like defects upto 636 μm in depth, which can be minimised using novel low abrasive air polishing powder.[6] The possibility of systemic absorption of NaHCO_3_ that raises blood levels of Na^+^ and CO_3_- probably through mucosal absorption, ingestion, inhalation, or combinations and development of marginal alkalosis as measured in venous blood exists and hence it is judicious not to use this device in individuals with Na restricted diet. Owing to the limitation of Prophy-Jet in sodium restricted diet individuals, non-sodium Prophy Powder, containing aluminium trihydroxide (Cavitron, Jet- fresh) instead of sodium bicarbonate has been introduced. The aerosols generated by air polishing may present an infection control hazard hence, preprocedural rinse is always recommended along with aerosol reduction devices (ARD). Subcutaneous emphysema which can occur whenever compressed air is employed intra-orally highlights the iatrogenic potential and reinforces the need to follow manufacturer's instructions appropriately.[7]

Recent introduction of Glycine Powder Air Polishing (GPAP) in removing sub-gingival biofilm abridge periodic sub-gingival instrumentation and serve as an alternative to conventional techniques that results in less gingival erosion alongwith 80% reduction in abrasiveness on root surface than hand instrumentation or NAHCO_3_ air-polishing.[8]
